# Oxygen therapy in interstitial lung disease – navigating benefit and burden

**DOI:** 10.1097/MCP.0000000000001288

**Published:** 2026-06-11

**Authors:** Sabina A. Guler, Yet H. Khor, Anne E. Holland, Magnus Ekström

**Affiliations:** a Department for Pulmonary Medicine, Allergology and Clinical Immunology, Inselspital, Bern University Hospital, University of Bern, Bern, Switzerland; b Respiratory Research@Alfred, School of Translational Medicine, Monash University, Melbourne; c Department of Respiratory and Sleep Medicine, Austin Health; d Institute for Breathing and Sleep, Heidelberg, Victoria; e Faculty of Medicine, University of Melbourne; f Department of Respiratory Medicine; g Departments of Physiotherapy and Respiratory Medicine, Alfred Health; h School of Translational Medicine, Monash University, Melbourne, Victoria, Australia; i Respiratory Medicine, Allergology and Palliative Medicine, Department of Clinical Sciences Lund, Lund University, Lund, Sweden

**Keywords:** hypoxemia, interstitial lung disease, oxygen therapy

## Abstract

**Purpose of review:**

Oxygen therapy is a key component of interstitial lung disease (ILD) management. This review summarizes the current evidence and explores how to balance its benefits and burdens in people with ILD.

**Recent findings:**

Hypoxemia frequently occurs in ILD and is associated with worse prognosis, but the extent to which oxygen therapy modifies outcomes remains uncertain. Observational findings suggest home oxygen therapy may reduce acute exacerbations and hospitalizations in people with ILD with a life-expectancy of more than 1 year. Ambulatory oxygen can improve symptoms and health-related quality of life in some patients; however, recent evidence indicates that portable oxygen concentrators may not improve daily oxygenation, symptoms, or physical activity. In patients with isolated exertional or nocturnal desaturation or significant respiratory symptoms with hypoxemia, careful consideration of individual benefit versus burden is essential within a shared decision-making framework. High-flow oxygen therapy is effective for acute respiratory failure, but its role in palliative care, pulmonary rehabilitation, and especially in the home environment, are areas of ongoing investigation.

**Summary:**

Oxygen therapy can support symptom relief in ILD, but its impact on long-term outcomes is unclear. High-quality evidence remains sparse, and advances in oxygen delivery technologies are needed to improve effectiveness while minimizing burden.

## INTRODUCTION

Interstitial lung diseases (ILDs) are characterized by a destruction of lung parenchyma through inflammation and fibrosis that causes a restrictive lung physiology and impaired gas transfer across the alveolocapillary membrane [1]. This leads to ventilation-perfusion mismatch that typically results in hypoxemia at rest and more pronounced during physical exercise. These contribute to dyspnea and a decline in exercise capacity in patients with ILD, with physical performance limitations in daily life and reduced quality of life [2].

Hypoxemia is common in ILD, with a cumulative incidence of 16% for resting hypoxemia and 40% for exertional hypoxemia after 5 years [2]. Older patients (age > 60 years) with idiopathic pulmonary fibrosis (IPF), with a higher BMI (BMI ≥25 kg/m^2^), low forced vital capacity (FVC < 50%-predicted) and low diffusing capacity of carbon monoxide (DLCO ≤35%-predicted) have a particularly high risk of developing resting (incidence 7%) and exertional hypoxemia (incidence 19%) within the next 6 months [3]. Furthermore, the development of hypoxemia is a risk factor for mortality beyond demographics and pulmonary function [2].

Home oxygen therapy aims to improve oxygenation, reduce dyspnea and improve exercise capacity to consequently improve health-related quality of life (HRQoL) and survival in patients with chronic lung diseases [4]. Oxygen therapy sources that can be applied in outpatient and home settings conventionally use low-flow devices and interfaces, most frequently nasal cannulas. Home oxygen sources include compressed gas cylinders, oxygen concentrators and liquid oxygen, which can be delivered using a continuous flow or a pulse-dosed delivery only during inspiration. Oxygen is prescribed at rest, during sleep and/or during exercise [4].

Even though patients with ILD frequently suffer from the consequences of hypoxemia, the evidence for oxygen therapy – specifically in this population – is sparce. In this review, we aim to summarize the current evidence and provide an overview of potential strategies on how to navigate oxygen therapy in people with ILD. 

**Box 1:**
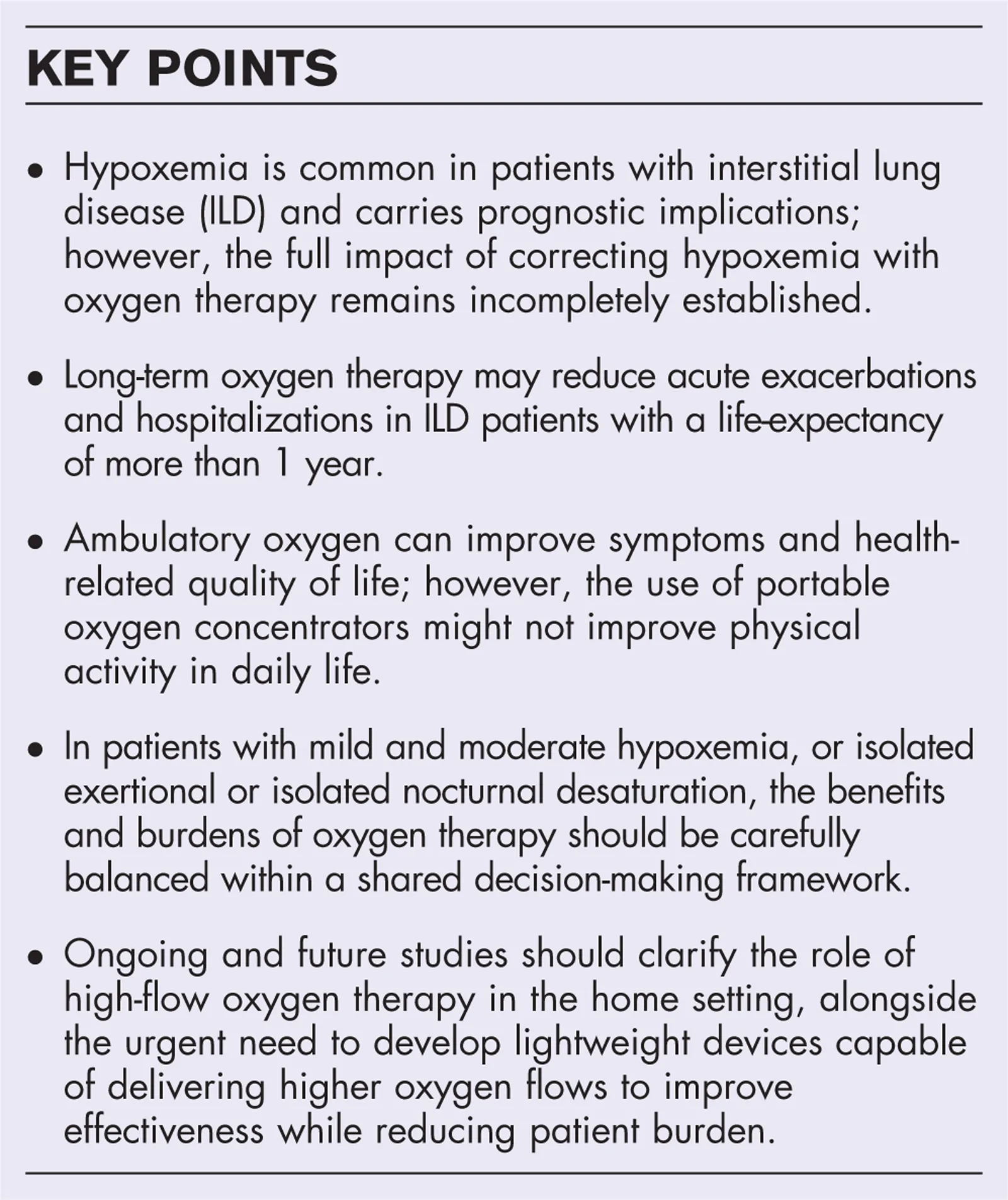
no caption available

## CURRENT EVIDENCE ON HOME OXYGEN THERAPY IN PEOPLE WITH INTERSTITIAL LUNG DISEASE

### Long-term oxygen therapy

Long-term oxygen therapy (LTOT) is recommended by international guidelines in people with ILD and chronic (longer than 3 weeks despite optimal management of the underlying disease) severe resting hypoxemia, defined as a partial pressure of arterial oxygen (*p*aO_2_) < 56 mmHg (7.4 kPa) or 56–59 mmHg (7.4–8 kPa) with signs of hypoxic organ damage including pulmonary hypertension or right heart failure [4,5]. These criteria were extrapolated from the two only efficacy trials of LTOT for severe resting hypoxemia, which were conducted in the 1970 s in 290 people with chronic obstructive pulmonary disease (COPD) [4,5]. These trials found that LTOT improved survival compared to no treatment [6], or treatment during the night only [7]. There are no published randomized clinical trials (RCTs) on the effect of LTOT specifically in people with ILD. An unpublished RCT (*n* = 62 participants) showed no difference in mortality with LTOT compared to air (no treatment); however, similar to the older trials in COPD, hospitalizations or patient-reported outcomes were not reported [8].

ILD is the second most common indication for initiating LTOT after COPD, accounting for approximately 15% of patients starting LTOT in current clinical practice, according to a population-based study from Sweden [9]. Recently, several clinical trials have reported findings with potential implications for oxygen therapy in people with ILD. The REDOX trial randomized 241 people with severe resting hypoxemia (14% with ILD, *n* = 34) to receive LTOT either prescribed for 24 or 15 h/day [10]. Compared to the 24 h/day group, LTOT 15 h/day resulted in a similar risk of hospitalization or mortality at one year, and there were no differences in patient-reported symptoms or HRQoL. Although it is possible that clinicians may have been reluctant to enroll some patients with ILD and the most severe resting hypoxemia, the characteristics of those included were similar to those starting LTOT nationally, including the severity of hypoxemia with mean resting *p*aO_2_ of 49 mmHg (6.5 kPa) on room air [10]. LTOT prescribed continuously for 24 h/day was also not shown to provide broader health economic benefits compared to LTOT 15 h/day [11]. These findings support the safety of being without LTOT for up to nine hours per day (with use recommended during the night) and suggest that LTOT can be tailored to the patient's life circumstances, activities and needs. However, the safety of this approach in people with extremely severe resting hypoxemia – which is more common in ILD than in COPD – and in those with significant comorbidities is less clear; therefore, management should be individualized.

In a large observational study including 2507 patients with ILD and severe resting hypoxemia, starting LTOT was associated with increased rates of exacerbations, hospitalization, and longer duration of hospital stay. Similar findings were observed in people starting LTOT for pulmonary hypertension [12^▪▪^]. Another observational study including 151 patients on LTOT (13% with ILD, *n* = 20) showed a high prevalence of side effects and adverse events related to the oxygen therapy [13]. While results were not evaluated separately in ILD and may partly reflect the underlying disease severity, a longer daily duration of oxygen use was associated with higher rates of side effects [14].

Taken together, LTOT is recommended for treating chronic severe resting hypoxemia in ILD, similarly to its use in COPD, with the aim of preventing hypoxemia-related organ damage and improving survival. However, emerging evidence challenges clinical net benefit of LTOT, calling for clinical trials to evaluate efficacy and to identify patients who are most likely to benefit.

### Nocturnal oxygen therapy

To date, no study has evaluated the long-term effects of nocturnal oxygen therapy in patients with ILD [15]. Previous studies in patients with COPD disease found no impact of nocturnal oxygen therapy on survival, progression to LTOT or HRQoL [16–18]. Nevertheless, nocturnal hypoxaemia affects approximately one in three patients with ILD, which is associated with the presence of pulmonary hypertension and increased mortality [19]. In a recent international stakeholder engagement study, the conduct of sleep assessments and eligibility criteria for nocturnal oxygen therapy were found to vary substantially across regions and jurisdictions [20^▪▪^]. The majority of patients with ILD and healthcare professionals with expertise in ILD, sleep medicine and/or oxygen therapy supported the importance of nocturnal hypoxaemia evaluation and management. Both groups highlighted the need for establishing therapeutic effects of nocturnal oxygen therapy on symptom burden, cognitive performance and HRQoL in patients with ILD. Furthermore, nocturnal oxygen therapy may have a role as an alternative therapy to continuous positive airway pressure therapy for the management of obstructive sleep apnoea (OSA) in patients with ILD, given the distinct underlying pathophysiological mechanisms of OSA in ILD compared to OSA without ILD [21,22].

### Ambulatory oxygen therapy

Ambulatory oxygen therapy (AOT) is oxygen delivered during exercise or activities of daily living, when the patient is walking freely. Delivering AOT during exercise testing in the laboratory consistently improves exercise performance (e.g. endurance time on a constant load test), however carryover of these benefits to use of AOT in daily life has been more difficult to demonstrate. The AmbOx trial, a crossover trial of AOT delivered with a portable cylinder during a 2-week treatment period (*n* = 84 participants) reported improvements in HRQoL and breathlessness compared to no oxygen [23]. More recently, the PFOX trial, an RCT with a 6-month follow-up period (*n* = 116 participants) did not demonstrate benefits of AOT delivered using a portable concentrator on daily physical activity, HRQoL or breathlessness. The PFOX trial highlighted the challenges of adherence, with 25% of participants choosing to cease AOT by 6 months, and the remainder using the device for less than 40 min/day on average [24^▪▪^].

Whilst some people with ILD report benefits of AOT for daily activity and reduction of breathlessness, others report unmet expectations for symptom relief and stigma associated with its use in public [25]. There is substantial dissatisfaction with AOT devices, which are perceived as heavy and bulky, delivering inadequate flow, having insufficient battery life, and being too costly [25]. As a result, shared decision-making is critical to support patients in weighing up the benefits versus potential burdens of AOT for their individual circumstances. Ideally, this would be supported by robust data indicating those patients with ILD who are most likely to benefit, but this is not yet available. In the AmbOx trial, the strongest predictors of choosing to continue AOT after 2 weeks were perceived reduction in breathlessness and perceived increase in walking ability, reinforcing the importance of early reassessment of the impacts of this complex therapy [23].

### Oxygen therapy for exercise training and pulmonary rehabilitation

The aim of delivering oxygen therapy during exercise training is to increase oxygen delivery to the exercising muscle, thus allowing higher exercise loads and maximising the training stimulus. Whilst the physiological rationale for use of oxygen during exercise training is strong with evidence suggesting improved physical performance of patients with ILD in laboratory settings [26^▪^], its impact on pulmonary rehabilitation outcomes has not been well documented and may be difficult to demonstrate over and above the substantial improvements conferred by pulmonary rehabilitation alone. An RCT of oxygen supplementation compared to air during exercise training in COPD, with blinding of participants and therapists, did not find additional benefits of training with oxygen [27]. The degree of exertional desaturation is generally greater in ILD than in COPD, so these results may not be directly applicable to people with ILD. There is increasing interest in the use of high flow oxygen therapy during exercise training in ILD, reflecting the challenges in correcting exertional desaturation. However, outcome data are not yet available [28]. Current guidance suggests that oxygen therapy should be used in pulmonary rehabilitation for patients who are already using LTOT or AOT [29]. Clinical practice at most pulmonary rehabilitation centres is to deliver oxygen therapy for patients with ILD and exertional hypoxemia to ensure safety, minimize symptoms during training, and perhaps enhance outcomes.

### High-flow nasal cannula oxygen therapy

Conventionally, oxygen therapy is delivered “low-flow” (typically 0.5–6 l/min) via conventional nasal cannula. In some patients with severe hypoxemia, the delivered oxygen flow is insufficient, and the conventional nasal cannula can cause upper airway dryness and nasal congestion. High-flow oxygen therapy (HFOT) delivered through high-flow nasal cannula (HFNC) provides heated, humidified oxygen at high flow rates (typically 20–60 l/min). This improves oxygenation and carbon dioxide wash-out by reducing anatomical dead space and reduces side effects including dehydration of the upper airway mucosa [30].

In hospital settings, there is emerging data for using HFOT in acute exacerbations of ILD (AE-ILD), although high-quality evidence remains limited. A retrospective single-centre cohort study of patients with AE-ILD and do-not-intubate orders found similar in-hospital and 30-day survival with HFNC compared to noninvasive ventilation (NIV), but fewer adverse events (e.g. skin damage), better tolerability, more continuous use, fewer discontinuations, and reduced need for sedatives with HFNC [31]. In a broader population with acute respiratory failure, a recent RCT did not show reduced 28-day mortality with HFNC versus conventional oxygen therapy, although intubation rates were lower-an important consideration in ILD given poor outcomes with mechanical ventilation [32].

In a home care setting, an ongoing Swedish RCT – recruiting patients with ILD and with COPD – is expected to provide evidence on the effect of HFOT versus standard LFOT on hospitalizations, death, symptoms, quality of life and healthcare utilization [33^▪^].

### Oxygen therapy in a palliative care setting

Oxygen therapy is frequently used to manage symptoms in patients with severe chronic lung diseases and in end-of-life care situations, and provides symptom relief in individual patients [34]. However, evidence is scarce and consequently, international guidelines are not able to provide recommendations for or against the administration of supplemental oxygen to reduce symptoms in people with serious respiratory illness [35]. A recent systematic review concluded that while supplemental oxygen reduces dyspnoea intensity during laboratory exercise testing, there is insufficient evidence to suggest an effect on dyspnoea in daily life or on HRQoL [36^▪^].

HFOT is frequently used in hospitalized patients with end-stage ILD and is associated with better symptom control and tolerability compared to conventional oxygen therapy [31]. Furthermore, a Japanese study of 177 ILD patients who died in hospital found that HFNC was associated with significantly higher bereaved family ratings of quality of dying and death compared to conventional oxygen therapy [37].

## NAVIGATING BENEFIT AND BURDEN OF OXYGEN THERAPY

As discussed above, patients with ILD may experience a range of potential benefits from oxygen therapy, although these are not universal. Reported benefits include improvement in shortness of breath, as well as a reduction in cough, chest tightness, dizziness and fatigue, alongside enhanced physical performance, sleep quality and overall quality of life [23]. It remains unsure whether the correction of hypoxemia leads to reduced risk or severity of pulmonary hypertension or improvement in cognitive function. In some patients, home oxygen therapy enables engagement in activities that would otherwise no longer be possible. Many of these benefits have primarily been described in qualitative studies [23,25], and have not consistently been demonstrated in quantitative studies.

**FIGURE 1:**
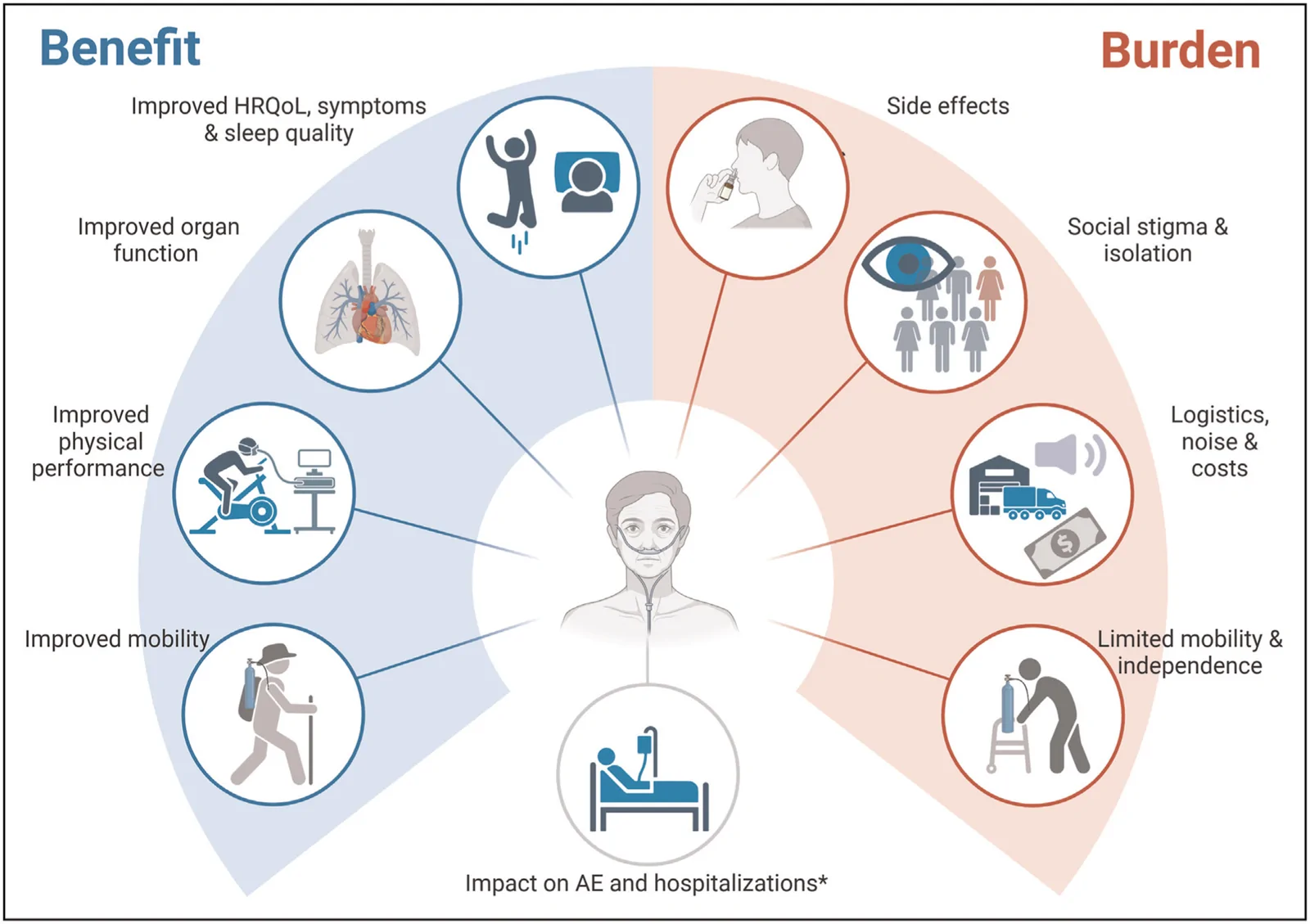
Potential benefit and burden from oxygen therapy experienced by patients with ILD. AE, acute exacerbation; HRQoL, health-related quality of life. ^a^The effect of oxygen therapy on acute exacerbations and hospitalizations may depend on the clinical context, with an observational study suggesting an overall increase in exacerbations and hospitalizations, but a decrease in these outcomes among ILD patients with a life expectancy exceeding one year. Created in BioRender. Guler, S. A. (2026) https://BioRender.com/f3pg768.

At the same time, oxygen therapy can impose a substantial burden on patients with ILD and their caregivers. Common challenges include upper airway side effects such as dryness and nasal congestion related to nasal cannula use, as well as logistical difficulties, including the inconvenience of carrying heavy equipment, noise of oxygen concentrators and the risk of tripping over oxygen tubes. Furthermore, some patients experience stigma or embarrassment when using oxygen in public. In less favourable scenarios, home oxygen therapy may lead to reduced activity, social isolation, and increasing frailty and dependence on others (Fig. 1) [23,25,38].

**FIGURE 2:**
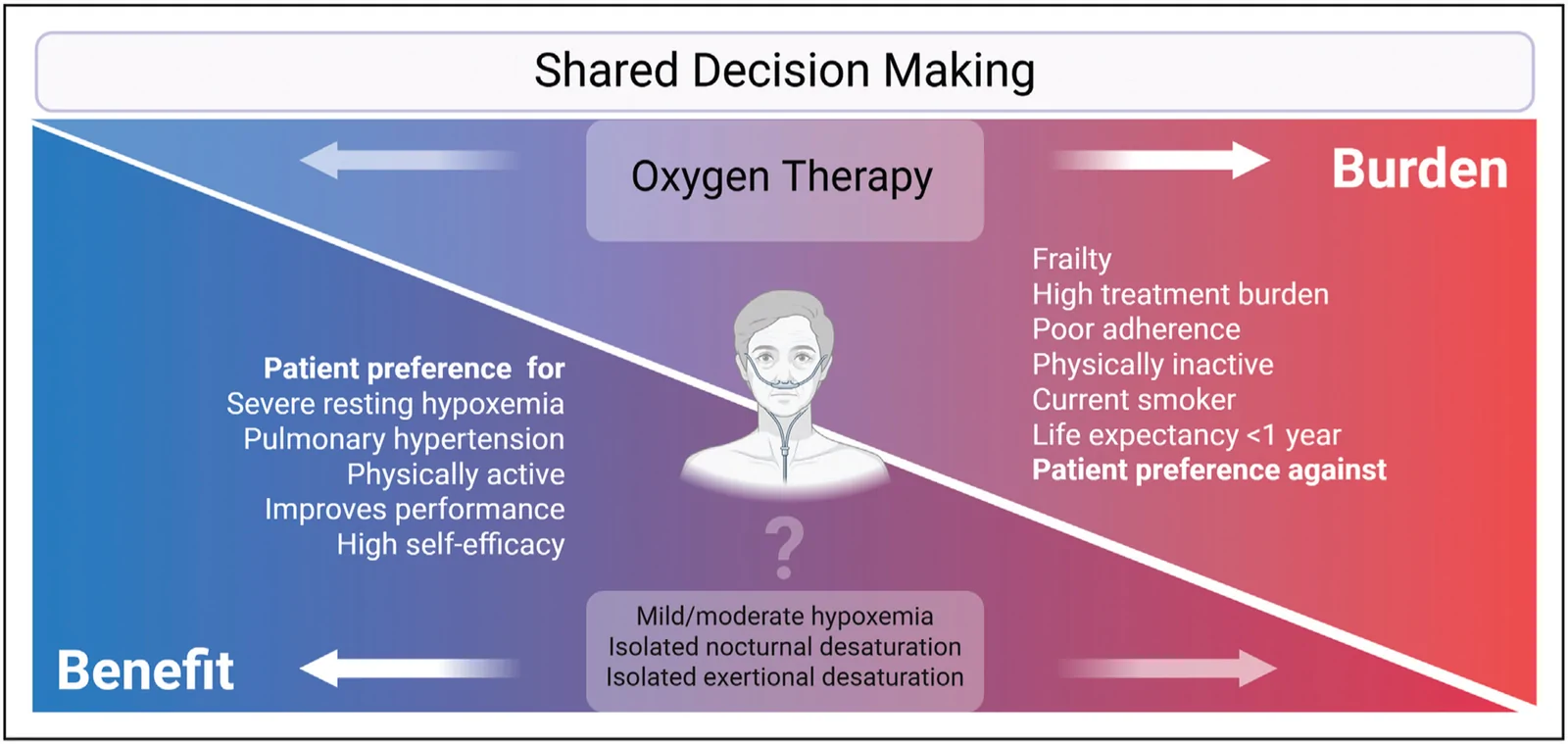
Balancing potential benefit and burden of home oxygen therapy for shared decision making. Individual patient features to incorporate into shared decision-making for the estimation of benefit versus burden from home oxygen therapy. Created in BioRender. Guler, S. A. (2026) https://BioRender.com/d4uji2x.

The balance of benefit versus burden is challenging to predict and likely varies across subpopulations. Patients with severe resting hypoxaemia are most likely to benefit and should generally be offered LTOT. Those with coexisting pulmonary hypertension may derive additional physiological benefit from improved oxygenation. In contrast, in frail patients or those with an already high treatment burden (polypharmacy, mobility aids) and limited life expectancy, the potential symptomatic benefit must be weighed carefully against the associated treatment burden, with a stronger focus on individual goals of care. To maximize benefit and minimize burden, careful selection of appropriate devices (e.g. lightweight portable concentrators, ambulatory liquid oxygen systems) is essential to preserve mobility and independence. Multidisciplinary support, including respiratory therapists, nurses and palliative care where appropriate, is equally important to address technical, physical and emotional challenges. Patient education is central, ensuring realistic expectations, well tolerated use and strategies to integrate oxygen therapy into daily life [39]. Ultimately, oxygen therapy in ILD should be individualized, with regular reassessment to align treatment with evolving clinical status and patient preferences (Fig. 2).

## CONCLUSION

Key challenges include limited ILD-specific evidence, heterogeneous responses, and the practical burden of oxygen therapy. Expert consensus underscores the need to move beyond extrapolation from COPD toward ILD-specific strategies, tailored to hypoxemia phenotype, comorbidities and individual goals. Conducting oxygen therapy trials in ILD remains difficult; adoption of standardized core outcomes [40^▪^], including patient-reported measures, adherence and cost-effectiveness, will be essential. Home monitoring may allow individualized titration for the prescription of home oxygen therapy. Technological innovation is urgently needed to deliver portable, high-performance and affordable systems. Alignment of reimbursement and policy with emerging evidence is also critical. Ultimately, patient-centred, multidisciplinary approaches should guide home oxygen therapy in people with ILD to maximize benefit while minimizing burden.

## Acknowledgements

*None.*

### Financial support and sponsorship

*None.*

### Conflicts of interest

*S.A.G. declares no conflicts of interest related to this work. Unrelated to this work, S.G. has received support from Boehringer Ingelheim, Roche, MSD, Puretech, Swiss Lung Ligue, Bernese Lung Ligue. M.E. declares no conflicts of interest related to this work. Unrelated to this work, M.E. has received a research grant from ResMed and personal fees from AstraZeneca, Boehringer Ingelheim, Novartis and Roche. Y.H.K. receives in-kind trial support from Air Liquide Healthcare and personal fees from AbbVie, Boehringer Ingelheim and Chiesi, outside of this work. A.E.H. reports in kind support from Air Liquide Healthcare and BOC Australia, unrelated to this work.*

## REFERENCES AND RECOMMENDED READING

Papers of particular interest, published within the annual period of review, have been highlighted as:

▪ of special interest
▪▪ of outstanding interest
